# One- and Two-Phase Software Requirement Classification Using Ensemble Deep Learning

**DOI:** 10.3390/e23101264

**Published:** 2021-09-28

**Authors:** Nouf Rahimi, Fathy Eassa, Lamiaa Elrefaei

**Affiliations:** 1 Computer Science Department, Faculty of Computing and Information Technology, King Abdul-Aziz University, Jeddah 21589, Saudi Arabia; feassa@kau.edu.sa; 2 Information System and Technology Department, Faculty of Computer Science and Engineering, University of Jeddah, Jeddah 21959, Saudi Arabia; 3 Electrical Engineering Department, Faculty of Engineering at Shoubra, Benha University, Cairo 11629, Egypt; lamia.alrefaai@feng.bu.edu.eg

**Keywords:** software requirement, functional requirement, non-functional requirement, classification, deep learning, ensemble, CNN, GRU, BiLSTM, LSTM

## Abstract

Recently, deep learning (DL) has been utilized successfully in different fields, achieving remarkable results. Thus, there is a noticeable focus on DL approaches to automate software engineering (SE) tasks such as maintenance, requirement extraction, and classification. An advanced utilization of DL is the ensemble approach, which aims to reduce error rates and learning time and improve performance. In this research, three ensemble approaches were applied: accuracy as a weight ensemble, mean ensemble, and accuracy per class as a weight ensemble with a combination of four different DL models—long short-term memory (LSTM), bidirectional long short-term memory (BiLSTM), a gated recurrent unit (GRU), and a convolutional neural network (CNN)—in order to classify the software requirement (SR) specification, the binary classification of SRs into functional requirement (FRs) or non-functional requirements (NFRs), and the multi-label classification of both FRs and NFRs into further experimental classes. The models were trained and tested on the PROMISE dataset. A one-phase classification system was developed to classify SRs directly into one of the 17 multi-classes of FRs and NFRs. In addition, a two-phase classification system was developed to classify SRs first into FRs or NFRs and to pass the output to the second phase of multi-class classification to 17 classes. The experimental results demonstrated that the proposed classification systems can lead to a competitive classification performance compared to the state-of-the-art methods. The two-phase classification system proved its robustness against the one-phase classification system, as it obtained a 95.7% accuracy in the binary classification phase and a 93.4% accuracy in the second phase of NFR and FR multi-class classification.

## 1. Introduction

Software requirement (SR) classification is carried out by software specialists to directly determine the requirements that they need or are interested in. For example, user interface designers are probably interested in “look and feel” requirements. The manual labeling or classification of each requirement to determine which category it belongs to is expensive and requires experts in different fields, which results in high costs. This provides motivation for finding a complete system with high performance to label and classify SR into functional requirements (FRs) or non-functional requirement (NFRs), as well as both FRs and NFRs into further classes [1].

The main classes of SRs are FRs and NFRs. FRs are defined according to the IEEE Standard Glossary of Software Engineering Terminology as a function carried out by a system or its components [2]. On the contrary, NFRs are one of the restrictions on system behavior [3]. Additionally, there are subcategories of both FRs and NFRs, as shown in Table 1 (FR subcategories [2]) and Table 2 (NFR subcategories [4]).

Deep learning (DL) has been adopted to automate and facilitate the tasks of software engineering (SE), such as maintenance, requirement extraction, and classification [6]. The results have been interesting, but some areas have not been extensively covered. SR classification plays a crucial role in software development as it facilitates the process and helps in avoiding further modification costs [7]. Moreover, it is not the matter of classification but the correct classification of SRs that is the crucial task in SE [3]. The correct classification of SRs into FRs or NFRs and the subcategories of NFRs and FRs are described as challenges due to many reasons related to the inconsistency among the different stakeholders and engineers, as they use different structures for the different classes, and the terms are not unified either. Thus, such automation of this classification is greatly needed [3].

Automation of the SR classification has been the main objective of a number of previous studies. Some of these studies used traditional automation tools and implemented simple software and tools, but there were disadvantages such as the complexity of the tools, incomplete extraction and classification (as NFRs were the main concern for the majority), unavailability, and low accuracy. ML or DL classifiers have been used in recent studies for SR classification as well. However, weaknesses have been observed among ML and DL solutions, such as incomplete solutions (as FRs have rarely been classified or ignored in some studies), accuracy issues, and availability problems. To overcome the weaknesses and the disadvantages of existing solutions, this study aimed to automate SR classification using DL models, along with ensemble methods, to form a complete model that conducted binary and multi-class classification for both FRs and NFRs.

The ensemble method is used more often than the DL method, although both are assumed to be good in different ways and do not make similar prediction errors. However, the results of the ensemble method are less sensitive to the specifications of training data. Moreover, it has been confirmed that the performance of a group of models is better than that of a single model [8]. Thus, ensemble learning was used in this research, mainly because it is an approved method to enhance the accuracy in a variety of scientific fields.

The use of ensemble models for SR classification is rare, especially in the field. In this work, different models were applied and the performance of the existing models and a new recent ensemble model were evaluated for first time using DL models, with accuracy per class as a weight. The results indicated its robustness in all experiments, achieving the best accuracy. Complete SR classification is also provided using one- and two-phase systems.

The contributions of this paper include:

1. Summary, categorization, and comparison of published research on ML or DL use for classifying SRs. The summarized categories involve classifying SRs into main classes, classifying FRs into multi-classes, classifying NFRs into multi-classes, and complete systems that classify SRs into main classes and FRs and NFRs into multi-classes.
2. Introduction of an ensemble learning framework based on DL models for classifying different types of SRs. The conducted experiments present comparative results between three different ensemble models—mean ensemble, accuracy as a weight ensemble, and accuracy per class as a weight ensemble—and show that accuracy per class as a weight ensemble learning classifier combining BiLSTM, LSTM, GRU, and CNN has a stronger capability to correctly predict different types of SRs.
3. Proposition of a two-phase system using the ensemble DL method. The first phase uses binary classification to classify SRs into FRs or NFRs, while the second phase classifies the output of the first phase (FRs or NFRs) into multi-classes. FRs are classified into six different labels: solution, enablement, action constraint, attribute constraint, policy, and definition. On the contrary, NFRs are classified into 11 different labels: availability, fault tolerance, legal and licensing, look and feel, maintainability, operability, performance, portability, scalability, security, and usability.
4. Proposition of a one-phase system using the ensemble DL method. The input is SRs and the output is 17 multi-classes of either FRs or NFRs (solution, enablement, action constraint, attribute constraint, policy, definition, availability, fault tolerance, legal and licensing, look and feel, maintainability, operability, performance, portability, scalability, security, and usability).
5. Investigation of the performance of each base DL classifier and a number of ensemble DL classifiers in each phase of the system.
6. To the best of our knowledge there is no complete two-phase system to classify SRs into FRs or NFRs, then to classify each FR and NFR into multi-classes using ensemble DL methodology. In addition, there is no such similar categorized review summary of the previous studies on classifying SRs.

The rest of the paper is organized as follows: Section 2 presents a review of the state of automated SR classification using the ML and DL methods. Section 3 displays the used methodology in detail, while Section 4 explains the details of the conducted experiments. Section 5 provides a comparative analysis, and Section 6 concludes this research. 

## 2. Related Work

This section summarizes previous work regarding the ML and DL methods that try to automate the classification of SRs into different categories: the binary classification of SRs into FRs or NFRs (Section 2.1), the multi-class classification of FRs (Section 2.2), the multi-class classification of NFRs (Section 2.3), and the complete systems that classify SRs into FRs or NFRs and both FRs and NFRs into subcategories (Section 2.4). 

### 2.1. Classifying SRs into Main Classes 

In [9], the authors assigned software requirements to multiple topics. The support vector machine (SVM) and multinomial naïve Bayes (MNB) models were used for classification purposes. Two German specifications by Mercedes-Benz were used, one of which was public and the other a confidential dataset. The results of these experiments recorded a maximum recall of 0.94 by the MNB classifier and a maximum precision of 0.86 by the SVM classifier.

In [10], a convolutional neural network (CNN) was used to classify text as either a software requirement or information. The Data Object Oriented Repository System (DOORS) dataset document, which was collected from 89 requirement specifications, was used to train and test the model. Using the CNN model to achieve the desired goal of classification recorded an accuracy of 81%, a precision of 0.73, and a recall of 0.89.

### 2.2. Classifying FRs into Multi-Classes 

The ensemble ML approach introduced in [11] aimed to classify FRs into six categories: Solution, enablement, action constraint, attribute constraint, definition, and policy. The ensemble method was an enhancement of accuracy as weight, as it used the accuracy per class as a weight to find the best classifier for each class. The ensemble combined five ML classifiers: SVM, naïve Bayes, logistic regression (LR), support vector classification (SVC), and decision tree. The model was trained and tested on a collected dataset that contained 600 FR statements with the same number of FRs from each class. The use of the most accurate three classifiers (SVM, SVC, and LR) outperformed the technique using all classifiers and achieved a 99.45% accuracy in 0.7 s. 

### 2.3. Classifying NFRs into Multi-Classes 

In [12], NFRs were categorized into approximately 14 categories, such as capacity, reliability, and security. The model was based on the K-nearest neighbor classifier and was tested on 11 different documents related to electronic health records (EHRs). The classifier was compared to the naïve Bayes and support vector machine classifiers and scored higher results with an F1 score of approximately 0.54. 

In [13], NFRs were categorized into different categories: Availability, look and feel, legal, maintainability, operational, performance, scalability, security, and usability. However, FRs were not categorized into deeper categories, and any software requirement that did not belong to any category was classified as an FR. The proposed classifier was designed to classify based on the frequency of terms that belonged to each category and calculated their weight using term frequency–inverse document frequency (TF-IDF). The classifier was trained and tested on 15 project documents that included 326 NFRs and 358 FRs. The results showed that considering only the top 15 frequent terms was the best technique, with a recall of 0.7669. 

In [14], the authors used the same dataset as that used in [13] but a different methodology. The naïve Bayes classifier was used, trained, and tested on more than 600 instances. To boost the classification, the expectation maximization algorithm was used to label untrained data. The overall accuracy was approximately 97%. 

In [15], the authors conducted a number of experiments to compare the classification of FRs and NFRs using an ensemble that combined two ML techniques: Random forest and gradient boosting. Raw data were in the of SQL and CSV formats. NFRs were classified into approximately 10 labels such as security, performance, and usability. FRs were not classified into further classes. The experiments aimed to measure the accuracy of FR and NFR classification. The results showed that for classifying NFRs, the gradient boosting algorithm performed better than random forest (0.826), while random forest was better at classifying FRs (0.591). 

In [16], SRs were categorized as FRs or NFRs using the naïve Bayes classifier. Then, NFRs were further categorized into classes such as security, availability, and scalability. The collected data for the experiment by the authors included 255 FRs, and the number of NFRs ranged from 10 to 67 per category. The classifier scored a 75% precision for NFRs. 

In [17], the PROMISE software engineering repository dataset was used to compare the performance of five ML algorithms (multinomial naïve Bayes (MNB), Gaussian naïve Bayes (GNB), Bernoulli naïve Bayes (BNB), K-nearest neighbor (KNN), support vector machine (SVM), stochastic gradient descent SVM (SGD SVM), and decision tree (Dtree)), along with different feature extraction techniques. Eleven categories were used to label NFRs into the categories of availability, legal, look and feel, maintainability, operational, performance, scalability, security, usability, fault tolerance, and portability. SGD SVM with TF-IDF scored the highest results of all of the classifiers, with a precision of 0.66, a recall of 0.61, an F1 score of 0.61, and an accuracy of 0.76. 

In [7], the authors also used CNN for classification purposes. The authors classified SRs into the categories of functional, availability, legal, look and feel, maintainability, operational, performance, scalability, security, usability, fault tolerance, and portability. The PROMISE corpus dataset was used for the experiment with the proposed DL model. The evaluation of the performance was measured using precession, recall, and F score; the results for these measures were 0.80, 0.785, and 0.77, respectively. 

In [18], a Master’s thesis was presented, which conducted experiments to perform a number of classifications: The binary classification of SRs into FRs or NFRs, the binary classification of NFRs (security-related and non-security-related), and the multi-label classification of NFRs (availability, legal, maintainability, operational, performance, scalability, look and feel, security, and usability). The PROMISE dataset was used to train and test the proposed CNN model. The results showed that the CNN classifier scored recall values of 0.945, 0.911, and 0.772 for the binary option of FRs or NFRs, the bidirectional encoder representations from transformers binary option of being security-related or not, and for NFR multi-label classification, respectively.

In [19], the authors classified NFRs into multi-classes using a specific type of tree classifier trained and tested using the Certification Commission for Healthcare Information Technology (CCHIT) dataset, achieving 93.92%. 

### 2.4. Complete System (Classifying NFRs and FRs into Multi-Classes)

In [20], the authors experimented with different classifications: Classifying SRs into FRs or NFRs, classifying NFRs into different categories (usability, security, operational, and performance), and classifying FRs into the categories of functions, data, and behavior. The model was a tuned (BERT) model named “NoRBERT”. Then, it was trained and tested on the PROMISE dataset for all experiments. The model recorded satisfactory F1 scores compared to existing state-of-the-art methods for the first classification objective (90% for FRs and 93% for NFRs). In NFR classification, it scored F1 scores of 76% and 92% for FR classification.

Analysis of the existing research on SR classification using the ML and DL methods, as summarized in Table 3, shows that different classifiers have been utilized, such as SVM, naïve Bayes, and CNN. However, binary classification or NFR classification into subcategories has been the main concern of the majority of studies, while FRs have attracted less attention and have rarely been classified into further classes. In addition, a complete system that classifies both FRs and NFRs into multi-classes has rarely been introduced, providing the motivation to conduct this piece of work.

## 3. Materials and Methods

### 3.1. Phases of Classification

**Figure 1:**
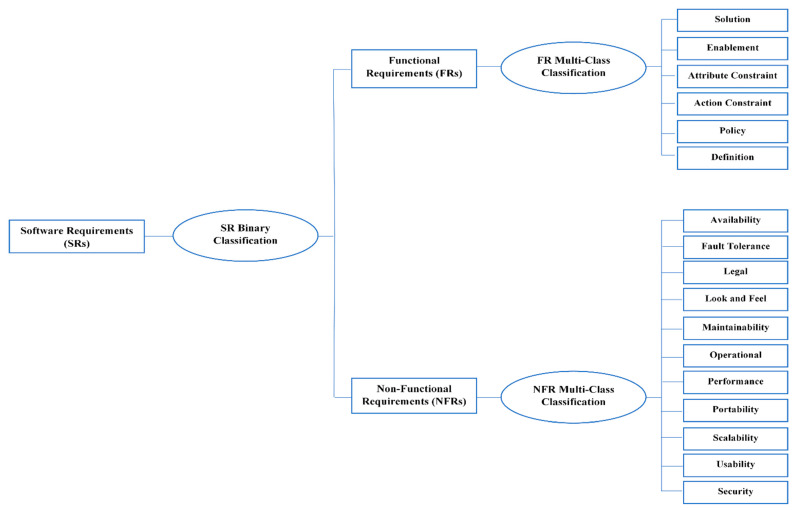
Phases of software requirement (SR) classification.

In this section, the phases of classifications are explained, as shown in Figure 1. First, SRs are classified into FRs or NFRs, and this is called binary classification. Second, FRs are classified into six different classes, as explained in detail in Table 1. The multi-classes of FRs include: solution, enablement, action constraint, attribute constraint, policy, and Definition. Third, NFRs are classified into 11 different classes, as summarized in Table 2. The multi-classes of NFRs include availability, fault tolerance, Legal, look and feel, maintainability, operational, performance, portability, scalability, usability, and security. In order to conduct these classifications, two experiments were conducted and are explained in the following sections.

### 3.2. Methodology

**Figure 2:**
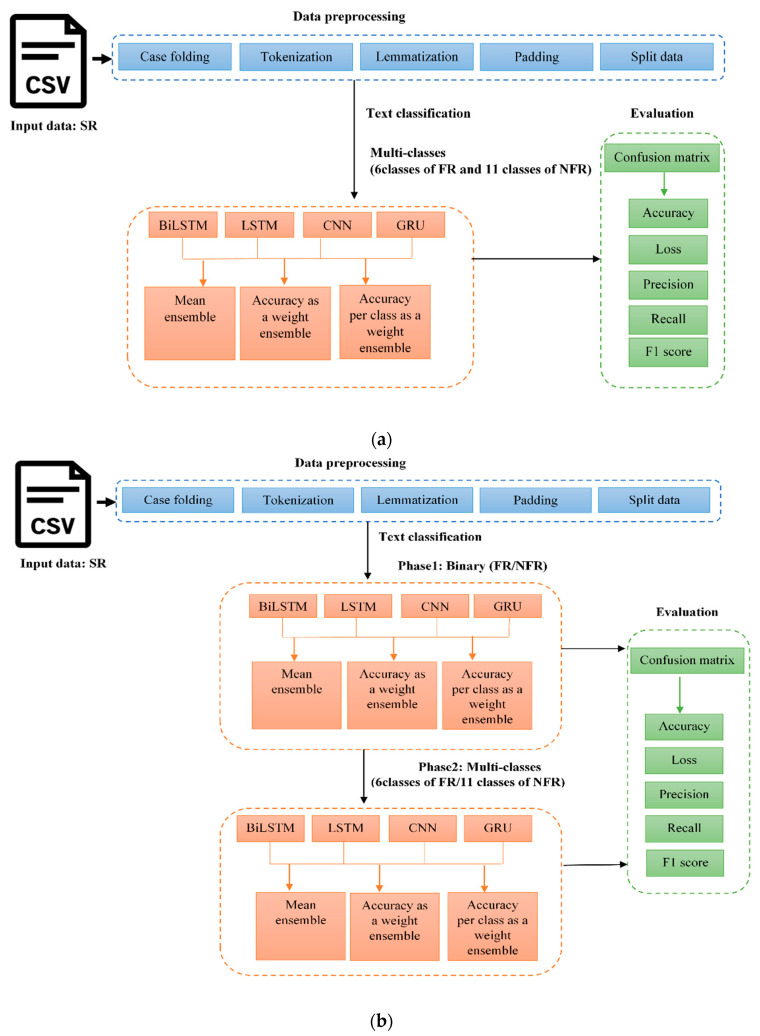
Proposed models (**a**) One phase (**b**) Two phases.

In this section, the proposed models are explained in detail. Figure 2 depicts the two models, which include the following phases, and the details are included in the subsequent sections:

- Data preprocessing;
- Text classification phase 1 (binary);
- Text classification phase 2 (multi-classes);
- Evaluation.

#### 3.2.1. Data Preprocessing

**Figure 3:**
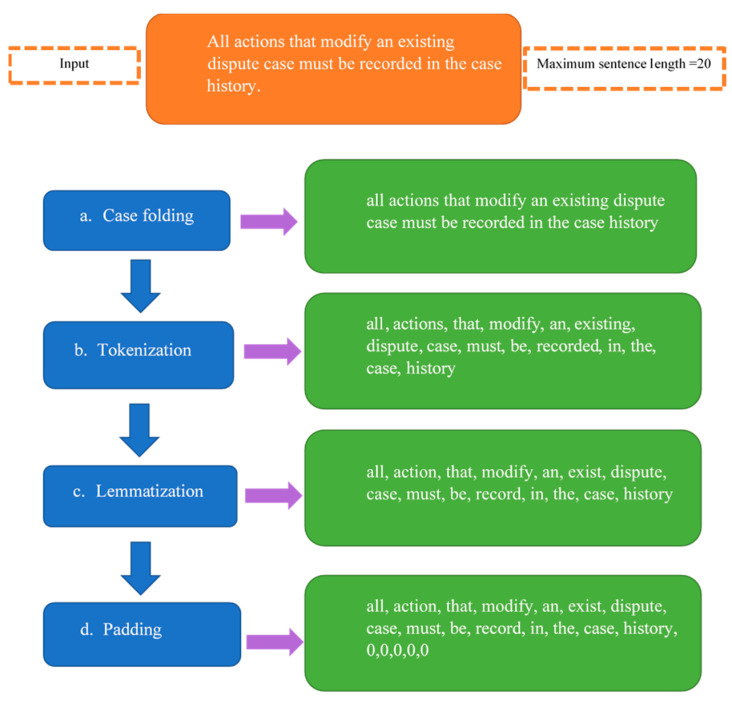
Preprocessing example.

This process is conducted only once at the beginning before phase 1 classification. Data preprocessing is a crucial step in DL models, as it affects the accuracy and quality of results. The preprocessing for the DL models was performed in a unified way. The preprocessing included four steps: (a) Case folding, (b) tokenization, (c) lemmatization, and (d) padding. The text was converted to lowercase in order to avoid having two dimensions for the same words [7]. Tokenization refers to converting the text to tokens of words [19]. Lemmatization means returning each word to its root and finding the correct base word for it, and it was selected because it outperforms stemming [21]. Finally, padding was used to unify the length of the sentences, since they had different numbers of words. This was carried out by finding the maximum length of the sentence and then adding zeros to the end of the sequence of tokens for any sentence that was shorter than the maximum length specified according to the input [20]. Algorithm 1 summarizes the preprocessing and Figure 3 gives an example of preprocessing of one SR from the dataset.
**Algorithm 1** (Data preprocessing step) **Input:  X:** A data stream of sentences inserted from a file.    **Y:** a label for the inserted sentences**Output:****    Train_data:** a section of the X and Y to train the algorithms.**    Validation_data:** a section of the X and Y to validate the algorithms.**    Test_data:** a section of the X and Y to test the algorithms.X < Tokenize sentences in X X < Remove spaces and stop words.X < Convector(X) // convert sentences into numbersY < Convector (Y) // convert labels (classes) into numbers// split data into training, validation and testing portions train_data (x, y), validation data(x,y), test_data(x,y) < **Split (X, Y)**

#### 3.2.2. Text Classification

A. Base DL Classifiers:

The following are the used base DL classifiers in both the binary and multi-class classifications:

- LSTM is a type of recurrent neural network (RNN) that uses memory blocks to solve the vanishing gradient problem in RNN using memory blocks. The model’s first layer is the input layer, which receives preprocessed data in a time step. Each component is passed to the embedding layer at first, which is represented to generate feature vectors. Then, the LSTM hidden layer follows the forward path only. LSTM has three main gates, namely, input, forget, and output gates, to control the cell state and update the weights [22].
- BiLSTM includes two hidden layers connected with both the input and the output. BiLSTM includes a forward LSTM layer and a backward LSTM layer to utilize the next tokens for learning information, and better predictions can be achieved. The best way to benefit from the BiLSTM is to stack LSTM layers. Forward layers are iterated from t = 1 to T. On the contrary, backward layers are iterated from t = T to 1 [23].
- CNNs involve producing local features by applying the convolutional concept [24]. Using filters with a width determined by the word embedding vector size, different vertical local regions allow different filter sizes of L = 2, 3, and 4. This is helpful for learning many features. Active convoluted results are used to generate feature maps with varied dimensions on the filters [25].
- GRU is type of RNN known for its fast conversions compared to LSTM. In addition, it requires fewer parameters [26]. It has two types of gates: An update gate and a reset gate. Since it has no memory to store information, it only deals with unit information. The update gate decides the amount of data to be updated and the reset gate decides the amount of past data to forget. If the gate is set to zero, it reads input data and ignores the previously calculated state [27].

B. Ensemble Models:

A number of ensemble methods are applied to the specified dataset (PROMISE). Then, the performance is compared. Details of each ensemble model are explained in the subsections of section B (B.1, B.2, and B.3).

B.1. Mean Ensemble:

This ensemble is also called simple averaging. The output is the average prediction from base classifiers that have equal weights [28]. The mean ensemble is explained in Algorithm 2.
**Algorithm 2** (Mean ensemble for base DL classifiers)**Input:**       **X:** Preprocessed data.          **Y:** labels of the sentences. **     NNAL(i):** Number of Neural Network algorithms used [BiLSTM, CNN, GRU, LSTM].**Output:****Mean_ Accuracy:** Represents the Mean ensemble model.***For each*** model in NNAL(i):  NNL(i) < fit (train_data (X, Y), validation(X,Y))  P(y′) < Predict (test data(X))  Result< Compare (p(y′), Y)   Conf(i)< Calculate (Confusion matrix)  Accuracy(i) < Result/Y*100 ***End******//Final prediction***Mean_Accuracy < mean(P(y′))/4

B.2. Accuracy as a weight:

This method is a combination of weight ensemble and simple averaging ensemble. The prediction of each model is multiplied by its weight, which is the accuracy in this case. Then, the average is calculated [29]. Algorithm 3 displays the accuracy as a weight ensemble.
**Algorithm 3** Accuracy as a weight ensemble for base DL classifiers**Input:**       **X:** Preprocessed data.          **Y:** labels of the sentences.**     NNAL(i):** Number of Neural Network algorithms used [BiLSTM, CNN, GRU, LSTM].**Output:****    W:** Weight for each NNAL which is its accuracy.**Voting_ Accuracy:** Represents the weight as accuracy ensemble voting model.***For each*** model in NNAL(i):  NNL(i) < fit (train_data (X, Y), validation(X,Y))  P(y′) < Predict (test data(X))  Result< Compare(p(y′), Y)   Conf(i)< Calculate (Confusion matrix)  Accuracy(i) < Result/Y*100   W(i) < Accuracy(i)***End******// Find the final prediction***V_result< Voting_algorithm (NNAL(i), W(i))Voting_ Accuracy < V_Result/Y*100

**Figure 4:**
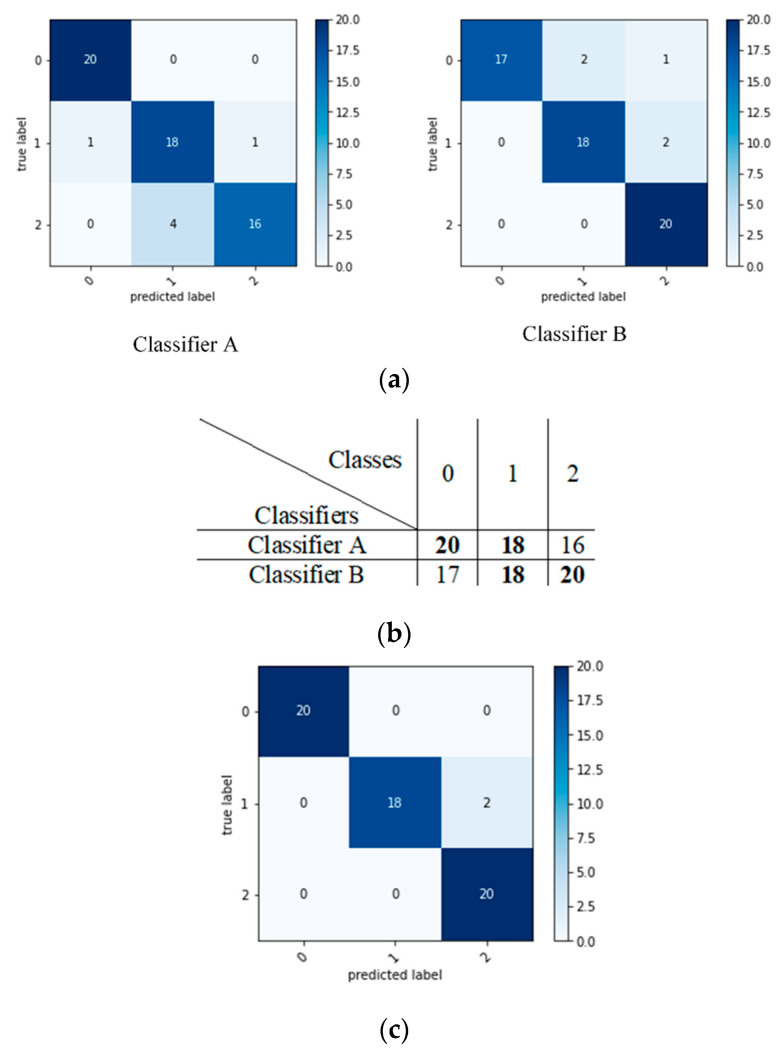
Example of accuracy per class as a weight. (**a**) confusion matrices for base classifiers; (**b**) matrix to store predictions of each class by each classifier (diagonals of confusion matrices); (**c**) confusion matrix of the final ensemble.

B.3. Accuracy per class as a weight:

This ensemble classifier is an enhancement of the voting ensemble, considering the accuracy as a weight. The enhancement consists of calculating the accuracy per class and passing it to the voting ensemble, rather than the overall accuracy, as other authors have done. This method of ensemble was proposed by [11], who applied the concept to ML classifiers. Figure 3 explains this ensemble method as having two base classifiers: Classifiers A and B. The type of classification is multi-class classification into class 0, 1, or 2. As per the example in Figure 4, the confusion matrices are created for base classifiers. Then, predictions for each class by each base classifier are stored as a matrix to be compared. For this example, for class 0, Classifier A prediction is better than Classifier B, so it is considered. For class 1, both classifiers have the same predictions, so one of them is selected randomly. Classifier B scores better prediction for class 2, resulting in it being considered. The better predictions for each class are considered to form the final confusion matrix. Algorithm 4 explains the accuracy per class as a weight ensemble model.
**Algorithm 4** Accuracy per class as a weight ensemble**Input: X:** Preprocessed data.   **Y:** labels of the sentences**   NNAL(i):** Number of Neural Network algorithms used [BiLSTM, CNN, GRU, LSTM].**Output:****W:** An array of weights assigned for each NNAL(i) **Voting_ Accuracy:** Represents the proposed ensemble voting model.***For each*** model in NNAL(i):  NNL(i) < fit (train_data (X, Y), validation_data (X, Y))  P(y′)  < Predict (test data(X))  Result < Compare(p(y′), Y)   Conf(i) < Calculate (Confusion matrix)  Accuracy(i) < Result/Y*100 ***End*****// Give a weight for each model**// combine the diagonals of all confusion matrices into one matrixConf_matrix < [[Conf (i. diagonal]]// find the maximum of each column that will represent the algorithm weightW < max_coloumn (Conf_matrix(i))V_result< Voting_algorithm (NNAL(i), W(i))Voting_ Accuracy < V_Result/Y*100

#### 3.2.3. Evaluation Methods 

Evaluation was conducted using different performance measures:

A. Confusion Matrix

The evaluation of each DL classifier was based on the confusion matrix, which summarized the correct and incorrect predictions of each classifier for each class, showing a ratio of correct predictions to the total predictions made by the classifier. The parameters that were required to calculate the other evaluation matrices were calculated through the confusion matrix. These parameters were the true positive (tp), false positive (fp), true negative (tn), and false negative (fn). The predicted classes are represented in rows, while actual classes are represented in columns [30].

B. Accuracy

Accuracy is one of the most important evaluation matrices, showing the overall performance of a classifier. The following formula was used to calculate the accuracy [31]:

(1) $$\mathrm{Accuracy}=\frac{\mathrm{tp}+\mathrm{tn}}{\mathrm{tp}+\mathrm{tn}+\mathrm{fp}+\mathrm{fn}}$$

where tp refers to true positive predictions, tn refers to true negative predictions, fp refers to false positive predictions, and fn refers to false negative predictions [31].

C. Loss

Loss was used to calculate the difference between predictions and true values. In the case that each input belonged to one category from the multiple categories, “sparse_categorical_crossentropy” was used. The following formula clarifies this calculation, where S refers samples, C refers to classes, and (SϵC) represents the samples belonging to class C [32]:

(2) Loss=−logp(SϵC)

D. Precision

The precision is the total of the positive prediction values and was calculated using the following formula [32]:

(3) $$\mathrm{Precision}=\frac{\mathrm{tp}}{\mathrm{tp}+\mathrm{fp}\;}$$

where tp refers to true positive predictions and fp refers to false positive predictions.

E. Recall

Recall is defined as sensitivity or the true positive rate and was calculated using the following formula [32]:

(4) $$\mathrm{Recall}=\frac{\mathrm{tp}}{\mathrm{tp}+\mathrm{fn}}$$

where tp refers to true positive predictions and fn refers to false negative predictions.

F. F1 Score

The F1 score is a relationship metric between recall and precision. It can be high only if both recall and precision are high. The F1 score was calculated using the following formula [32]:

(5) $$F1-\mathrm{Score}=2*\frac{\mathrm{Precision}*\mathrm{Recall}}{\mathrm{Precision}+\mathrm{Recall}\;}\;$$

## 4. Experiment Details

### 4.1. Hardware and Software Details

Python 3.6 was used for implementing the base and ensemble classifier. We used the PyCharm tool, which is user-friendly, and its dataset allows for easy uploading. This is true for various dataset file formats, such as CSV. 

Scikit learn was also chosen, as it includes many libraries that can facilitate building ML classifiers and calculating the factors used in the evaluations. Ensemble models are widely supported by all types of classifiers.

The computer used was an ASUS Laptop, with an x64 Inter(R) Core (TM) i7-9750H processor and 17.0 GB of RAM, running a 64-bit Windows Operating System.

### 4.2. PROMISE Dataset

This is a widely used dataset for SR classification in most previous work. It consists of FRs and NFRs labeled into 11 different categories, as explained in Table 1, with a different number of statements in each class. The content of the found and used version of the PROMISE dataset is detailed in Table 4. 

The FRs were labeled into six classes by the authors to be used in the experiments, containing different numbers in each class, as shown in Table 5. 

The split of the dataset into training, validation, and testing was unified for all systems and experiments to 35% training, 35% validation, and 30% testing. An imbalanced dataset has different solutions to form a balanced dataset for experiments. The selected solution according to the available dataset was random oversampling. This technique duplicates minority classes in training datasets and is repeated until satisfaction of class distribution is reached [32].

### 4.3. One-Phase Classification System

A. Training

This system classifies SRs into one of the FR or NFR classes (17 classes) directly without passing the phase of binary classification at the beginning. The validation dataset was used to ensure that there is no overfitting in each base DL classifier. The hyperparameters were left to the default and some were justified to the values that help to avoid overfitting. The Adam optimizer was used with a 0.001 default learning rate. The suitable number of epochs was 30 with batch size of 50. Dropout was justified to 0.2 for all classifiers. The architecture of each base DL model is summarized in Table 6, Table 7, Table 8 and Table 9.

**Figure 5:**
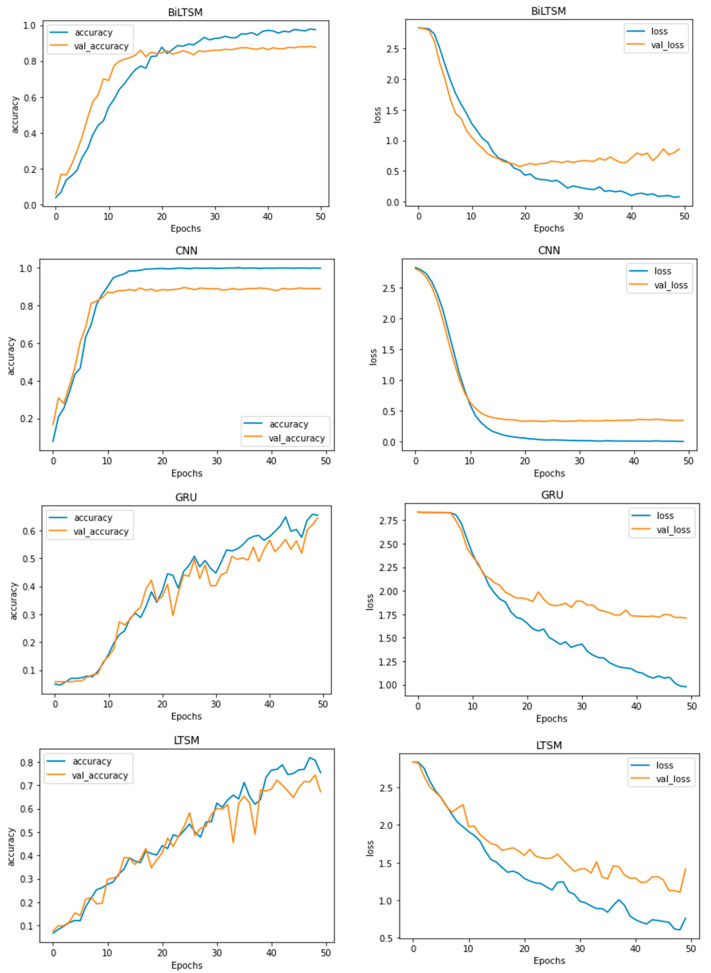
Accuracy–loss curves for the DL models in 60 epochs.

Figure 5 shows the accuracy and loss for DL models during 60 epochs to find the optimal number of epochs. As it was noticed that 30 epochs is optimal for all DL models, this number of epochs was selected for all experiments.

B. Test Results and Discussion

**Figure 6:**
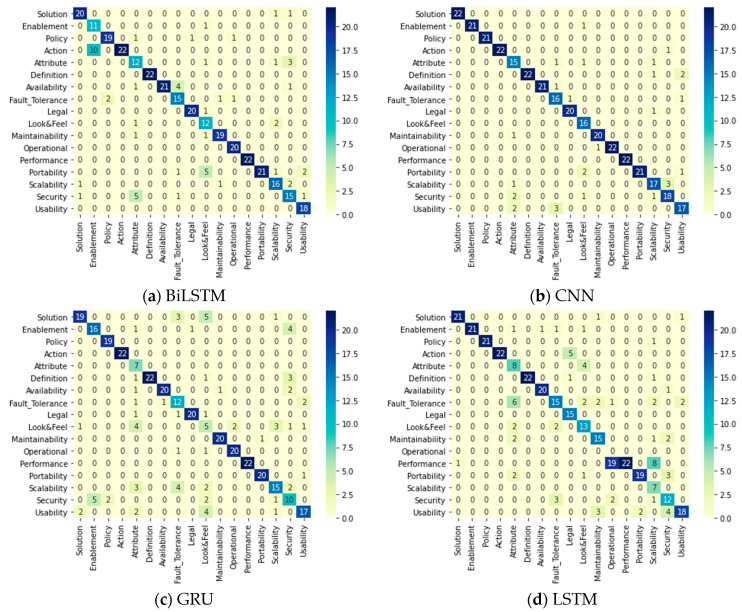

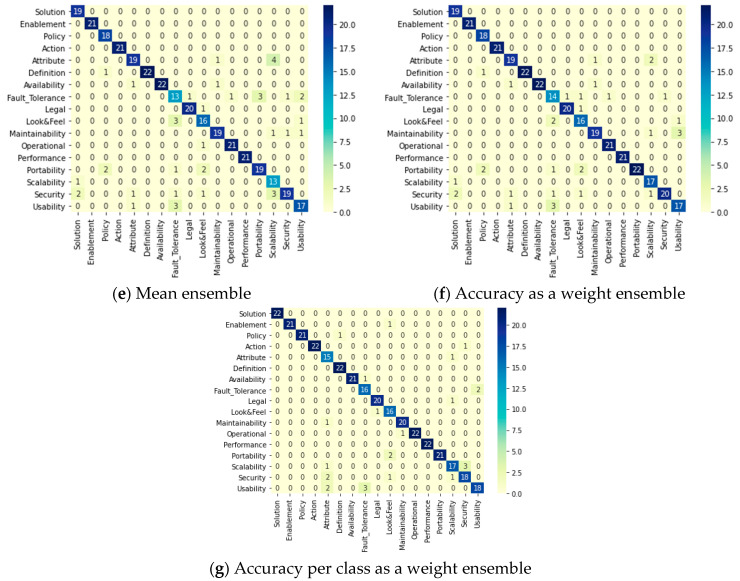
One-phase system confusion matrices for the DL classifiers.

Table 10 summarizes the evaluation parameters in the testing phase for SR multi-class classification. The base DL models reached a 91% accuracy, a 0.92 precision, a 0.92 recall, and a 0.92 F1 score by the CNN model. The applied ensemble methods reached a 92.56% accuracy, a 0.93 precision, a 0.93 recall, a 0.93 F1 score by accuracy per class as a weight ensemble method. The confusion matrices of all models and methods applied on the PROMISE dataset are displayed in Figure 6, where it can be noticed that the most accurate predictions for all classes were reached by the accuracy per class as a weight ensemble method. The ensemble method was able to enhance the accuracy of the SR classification, but the authors would prefer to examine the application phases of classification on the PROMISE dataset to find the best approach—either a one- or two-phase system. 

### 4.4. Two-Phase Classification System

#### 4.4.1. Binary Classification of SRs into an FR or NFR Phase

A. Training

This system classifies SRs into FRs or NFRs as the first phase, and the preprocessed and binary-classified SRs are then passed to the second phase for multi-class classification. The validation dataset was used to ensure that there was no overfitting in each base DL classifier. The hyperparameters were left as default, and some were justified to values that help to avoid overfitting. The Adam optimizer was used with a 0.001 default learning rate. The suitable number of epochs was 30 with a batch size of 50. Dropout was justified to 0.2 for all classifiers. The architecture of each base DL model is summarized in Table 11, Table 12, Table 13 and Table 14.

B. Test Results and discussion

**Figure 7:**
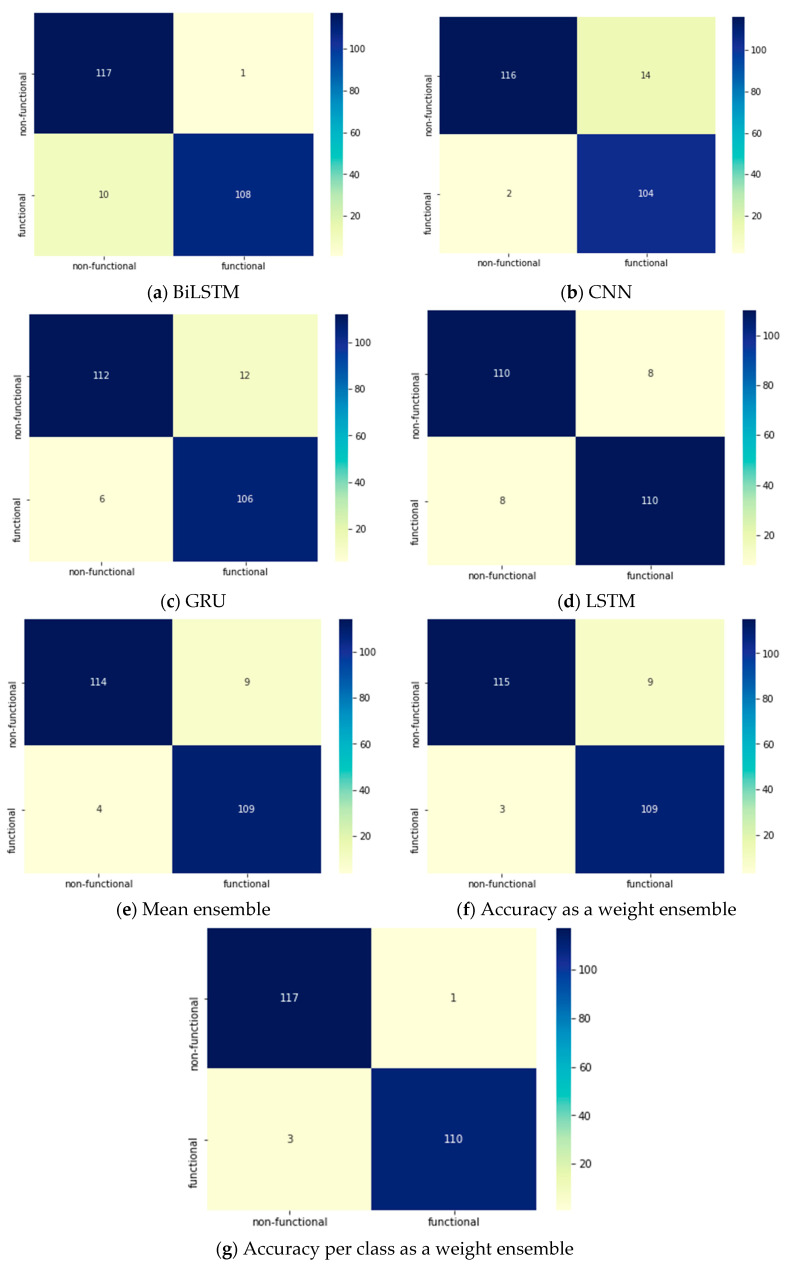
Two-phase system: Confusion matrices for the DL models in the binary classification phase.

Table 15 displays the performance attributes of each DL model and ensemble method applied to the PROMISE dataset during the binary classification phase of SRs. The DL models were able to reach close results of 94.49%, 93.22%, 93.22%, and 92.37% by BiLSTM, LSTM, CNN, and GRU, respectively. However, in terms of the ensemble methods, the accuracy as a weight and accuracy per class as a weight were able to enhance the accuracy by achieving 94.9% and 95.7%, respectively. The other performance evaluation attributes achieved 0.96 by using accuracy per class as a weight ensemble method. Figure 7 shows the confusion matrices for all of the DL models and ensemble methods and clarifies that the best prediction accuracy was achieved by accuracy per class as a weight ensemble method.

#### 4.4.2. Multi-Class Classification Phase of NFRs and FRs into 17 Classes

A. Training

Training the model to handle FRs and NFRs requires different parameters to be tuned and justified to achieve acceptable results, as the model should be trained to modify itself to search FR classes if the input from the first phase (binary classification) is FRs and to search in the NFR classes if the input from the first phase is NFRs. Table 16, Table 17, Table 18 and Table 19 summarize the parameters of the base DL models in the training phase for FRs, and Table 20, Table 21, Table 22 and Table 23 summarize the parameters of the base DL models in the training phase of NFRs.

B. Test Results and discussion

**Figure 8:**
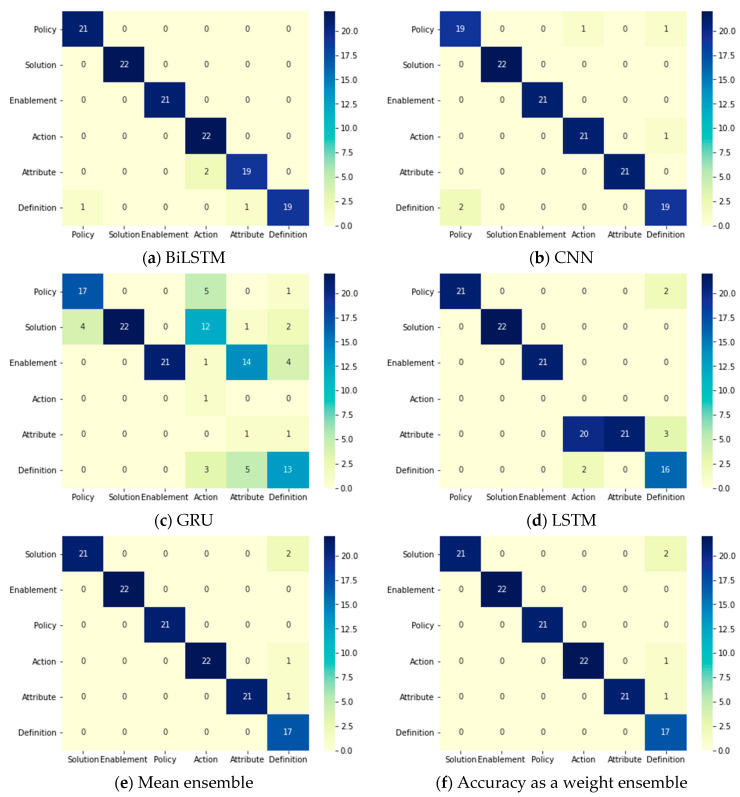

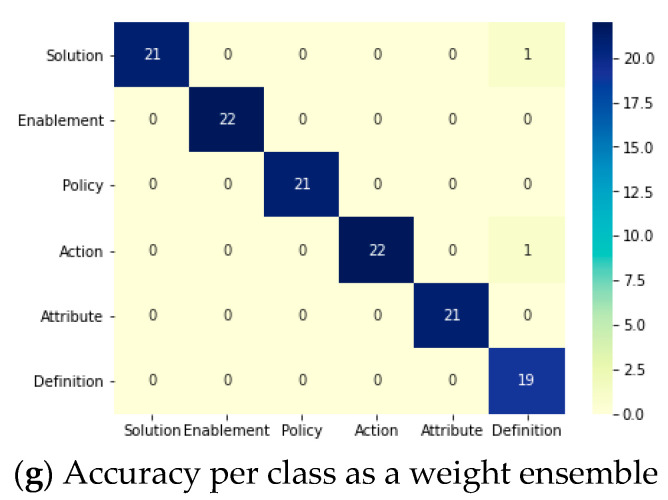
Two-phases system: confusion matrices of the FR DL classifiers in the multi-class classification phase.

Table 24 clarifies the results of all of the used DL models and ensemble methods that have been applied to classify FRs from the PROMISE dataset after being preprocessed and binary classified by the same model in the previous phase. The base DL models performed successfully in the case of BiLSTM and CNN, as they achieved accuracies of 96.8% and 96.0%, respectively. However, the LSTM and GRU models only achieved accuracies of 78.91% and 58.59%, respectively, indicating lower performance than the other DL models. The ensemble methods achieved equal accuracy percentages as BiLSTM model (96.8%). All confusion matrices for all DL models and ensemble methods are displayed in Figure 8. It can be clearly seen that the best predictions for all FR classes were achieved by accuracy per class as a weight ensemble method. 

**Figure 9:**
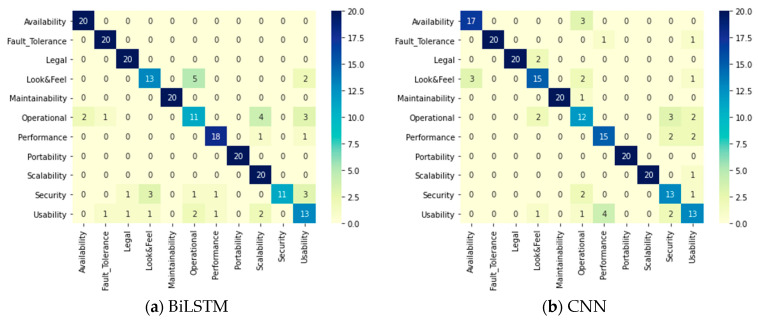

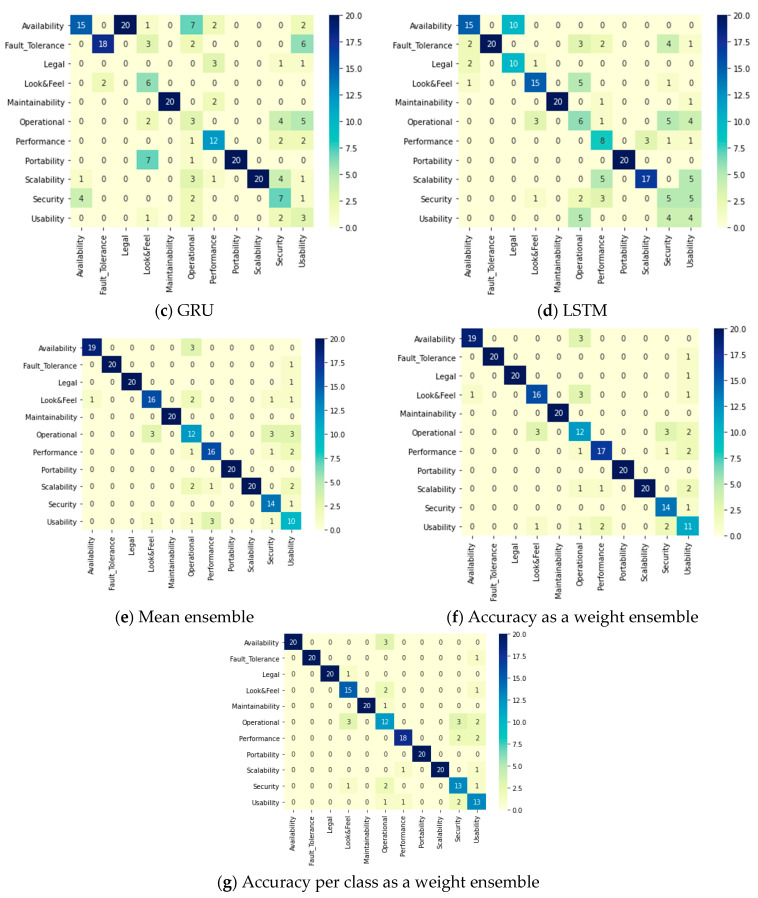
Two-phase system: Confusion matrices of the NFR DL classifiers in the multi-class classification phase.

Table 25 displays the performance of all the applied classifiers: DL models and ensemble methods in the NFR multi-class classification phase, which comes after the binary phase. In terms of the base DL models, BiLSTM and CNN achieved accuracies of approximately 83%, while LSTM and GRU achieved accuracies of 63% and 56%, respectively. All ensemble methods successfully improved the accuracy. However, the best accuracy was achieved by accuracy per class ensemble method (86.5%). The recall and F1 score were 0.86 and 0.87, respectively, but the precision was 0.89. Moreover, Figure 9 displays the predictions of each DL model and ensemble method with a clear indication that accuracy per class as a weight ensemble method has the maximum prediction values among the others.

#### 4.4.3. All Together Two-Phase Classification System 

Table 26 displays the final results of the evaluation parameters for the two-phase classification system all together. All DL models and ensemble methods performed better when using the two-phase classification system, as the binary phase facilitates the multi-class classification phase because it carries out the preprocessing and informs each model to justify itself to either focus on FR or NFR classes. Better evaluation parameters were achieved, but the accuracy per class as a weight ensemble recorded the best parameter values among the other DL models and ensemble methods, and even compared to itself in one-phase classification system, as it scored a 93.4% accuracy, a 0.94 precision, a 0.94 recall, and a 0.93 F1 score. 

## 5. Comparative Analysis

In order to prove that the two-phase classification system using the ensemble method based on DL models has an impact on the field and shows the best performance among the existing state-of-the-art methodologies, it was compared experimentally with other approaches applied on the same dataset (PROMISE). Table 27 summarizes the results of previous studies that have applied their suggested methodology using ML or DL on PROMISE dataset and the proposed system. In [7], the authors achieved a 0.80 precision in classifying SRs into 11 classes of NFRs or FRs if it does not belong to one of the eleven classes using the CNN model. However, in [19], the authors started by binary classification of SRs, recording a 0.94 F1 score. Then, NFRs were classified using a smaller number of classes, as used in [20], achieving a 0.91 F1 score, but NFRs were also classified into security-related or non-security-related, with a 0.77 F1 score. In [17], the authors classified NFRs into 11 classes, scoring a 76% accuracy. Only [20] provided a complete classification system. SRs were classified into FRs or NFRs with an average F1 score of 91.5%. Then, NFRs were classified into four classes only: Usability, security, operational, and performance, with a 76% F1 score. FRs were also classified into FRs or data behavior, with a 92% F1 score. Although a complete system was presented, the proposed classification systems herein outperform this system. Moreover, it only classifies NFRs into four classes, while the proposed classification system here classifies NFRs into 11 classes. In addition, using the previously mentioned system, FRs are classified into two classes, but our proposed system classifies FRs into six classes. The main comparison attribute is the performance, as the proposed one-phase classification system achieved a 92.56% accuracy, which has not been achieved before. Moreover, the proposed two-phase classification system achieved a 95.7% accuracy in the binary phase, which is the highest among all previous studies’ results in binary classification. In the second phase of the multi-class classification, it scored a 93.4% accuracy. This is clear confirmation that the proposed systems are better than others, with the two-phase classification system being better than the one-phase classification system.

Comparing the proposed systems with the existing state-of-the-art methods proved that it improved the accuracy and outperformed the other approaches. The conducted experiments proved the success of the proposed systems using the ensemble DL approach in the binary classification of SRs into FRs or NFRs, subcategories of NFRs, and subcategories of FRs, which have received the least attention from authors previously. 

## 6. Conclusions and Future Work 

This study utilized ensemble DL methods to develop one- and two-phases classification systems to classify SRs in different ways: Binary and 17 multi-class classification of FRs and NFRs. The main objective was to enhance the accuracy and availability. The BiLSTM, CNN, LSTM, and GRU ensemble models were used as base DL models. Compared to a number of existing state-of-the-art ensemble methods, it was found that accuracy per class as a weight ensemble method was the best among the other ensembles. The proposed systems are considered from the few complete SR classification systems. The systems provide binary and multi-class classification with a high accuracy, as the one-phase system achieved a 92.56% accuracy, while the two-phase classification system reached 95.75% in the binary phase and 93.4% in the multi-class classification phase. Thus, it can be concluded that both of the proposed systems have leading performance results, with the two-phase classification system being better than the one-phase classification system. 

The goal of this work was to introduce and provide SR classification systems that would help software engineers, developers, and analysts to produce complete SRs to build reliable software systems. These SR classification systems are based on the ensemble approach, which was applied using DL models for the first-time. These systems are complete systems, as they include binary classification, multi-class classification of FRs, and multi-class classification of NFRs, with a maximum number of classes. The accuracy is high, and its robustness was confirmed compared to the existing state-of-the-art approaches. 

The limitations of the proposed model and the classification systems that are based on it include supporting one language, as it could support multiple languages. Moreover, it can be upgraded to deal with a whole document that is unstructured and can extract sentences of SRs from it instead of dealing with extracted structured sentences.

## Figures and Tables

**Table 1:** Six different classes of functional requirements (FRs) [4].

Class	Definition	Example
Solution	Includes actions that are expected to be carried out by the system or different users.	“The system shall display completed worklist items to the Lab Manager.”
Enablement	Includes the capabilities offered by the system to different users. Subsystems that offer these capabilities are optionally specified.	“The Lab Manager shall be able to create worklist items.”
Attribute Constraint	Constraints on attributes or entity attributes are specified through this class.	“Search options must always be one of the following: Price, destination, restaurant type, or specific dish.”
Action Constraint	Allowed and non-allowed actions by the system or the subsystems displayed using this class.	“The loan subsystem may only delete a lender if there are no loans in the portfolio associated with this lender.”
Policy	This class is responsible for specifying all policies that have been decided for the system.	“A loan is not computed in more than one bundle.”
Definition	All entities are defined using this class.	“The expected profit of a fixed-rate loan is defined as the amount of interest received over the remaining life of the loan.”

**Table 2:** Non-functional requirement (NFR) subcategories [5].

Class	Definition	Example
Availability	Related to users’ accessibility to a system at a specific time.	“The product shall be available 24 h per day, seven days per week.”
Fault Tolerance	Determines the degree to which the system or product operates in the absence of hardware or software faults.	“100% of saved user preferences shall be restored when the system comes back online.”
Legal and Licensing	Determines the licenses and certificates that the system needs to obtain.	“All actions that modify an existing dispute case must be recorded in the case history.”
Look and Feel	Specifies the appearance and style.	“The website design should be modern, clean, and concise.”
Maintainability	Effectiveness and efficiency of a system modified by maintainers.	“Application updates shall occur between 3:00 a.m. and 6:00 a.m. CST on Wednesday morning during the middle of the NFL season.”
Operability	How easy to operate and control a system through its attributes.	“The product shall run on the existing hardware for all environments.”
Performance	Related to required resources at a specific condition.	“A customer shall be able to check the status of their prepaid card by entering in the PIN number in under 5 s.”
Portability	The effectiveness and efficiency in transferring the system from one hardware, software, or environment to another.	“The product is expected to run on Windows CE and Palm operating systems.”
Scalability	The degree to which a system adapts effectively and efficiently from one hardware, software, or environment to another.	“The system shall be able to handle all of the user requests/usage during business hours.”
Security	Includes the protection of personal data and authorized access.	“The product shall provide authentication and authorization.”
Usability	The level of efficiency and effectiveness of a system that can be used by specific users to reach specific goals with satisfaction.	“The product shall be installed by an untrained realtor without recourse to separately printed instructions.”

**Table 3:** Summary of related work.

Reference # Year	Classification		Methodology	Dataset	Results	Advantages	Disadvantages
	Input	Classes					
1. Classifying SRs into Main Classes							
[9] 2013	Software requirements (SRs)	Topics	Support Vector Machine (SVM) and Multinomial Naïve Bayes (MNB)	Two German specifications by Mercedes-Benz (public and confidential)	Recall: 0.94 (MNB) Precision: 0.86 (SVM)	Reliable results. Improves technical review to enhance SRs.	No enough training data.
[10] 2017	Text	Requirement OR Information	Convolutional Neural Network (CNN)	DOORS document database	Accuracy: 81% Precision: 0.73 Recall: 0.89	One of the first studies that used CNN.	Does not point to wrongly classified input.
2. Classifying FRs into Multi-Classes							
[11] 2020	Functional requirements (FRs)	Solution Enablement Action constraint Attribute constraint Definition Policy	Ensemble (accuracy per class as a weight) includes support vector classification, support vector machine, decision tree, logistic regression, and naïve Bayes	Collected dataset that has 600 FR statements with 100 FRs from each category	Accuracy: 99.45% Time: 0.07 s	Novel ensemble approach. New introduced FR dataset.	Limited to FRs.
3. Classifying Non-Functional Requirements (NFRs) into Multi-Classes							
[7] 2017	SRs	Functional Availability Legal Look and feel Maintainability Operational Performance Scalability Security Usability Fault tolerance Portability	CNN	PROMISE corpus	Precession: 0.80 Recall: 0.785 F-measure: 0.77	SRs classified into 12 classes using DL. SRs prepared simply using the proposed reprocessing method. DL evaluated to support RE.	Limited to CNN only, while other models could be used.
[18] 2018	1. SRs 2. NFRs 3. NFRs	1. FRs NFRs 2. Availability Legal Maintainability Operational Performance Scalability Look and feel Security usability 3. Security Not Security	CNN	PROMISE corpus	1. F1 score: 0.945 2. F1 score: 0.911 3. F1 score: 0.772	Utilizing word embedding in vectorization leads to improvement in SR classification.	Various aspects unexplored in the method and validation.
[19] 2014	SRs	FR Access control Person authentication Security encryption Decryption Audit control Automatic Logoff Integrity controls Unique user Identification Transmission Encryption Decryption Emergency Access procedure Transmission Security	WekaClassifiersTrees REPTree J48	Certification Commission for Healthcare Information Technology (CCHIT) dataset	Accuracy: 93.92%	ML algorithms combined to solve 2 RE problems, and more problems can be solved.	Additional components required to solve RE problems with Tracelab.
[12] 2013	NFRs	14 NFR classes such as: Capacity Reliability Security	K-nearest neighbor classifier	11 documents related to electronic health records (EHRs)	F1: 0.54	Ability to extract relevant NFRs from documents.	Limited to NFRs. Specific data field.
[13] 2007	SR	FRs Availability Look and-feel legal Maintainability Operational Performance Scalability Security Usability	Classification based on key words for each category, along with finding the weight based on frequency for each keyword used by TF-IDF	Labeled 326 NFRs and 358 FRs from 15 different projects	Considering only the top 15 keywords for each category scored the highest Recall: 0.7669	Provided an incremental approach for training a classifier in new domains.	More efforts to improve stopping conditions. Run time needs to be improved to be used by a real analyst.
[14] 2010	SR	FRs Availability Look and feel Legal Maintainability Operational Performance Scalability Security Usability	Naïve Bayes classifier	Labeled 326 NFRs and 358 FRs from 15 different projects	Accuracy: 97%	Feasibility of using semi-supervised learning in NFR classification.	Dataset is limited to the top-ranked examples.
[15] 2019	SR	FRs NFRs categorized into 10 categories such as: Security Performance Usability	Ensemble method consists of random forest and gradient boosting	Text files in a format of SQL and CSV files	NFR accuracy: 0.826 (gradient boosting) FR accuracy: 0.591 (random forest)	Highly accurate algorithms for SR classification.	Limited study due to limited data. Could not determine the number of SRs in a sentence. Could not read input from .docx and .txt files.
[16] 2010	SR	FRs NFRs categorized into categories such as: Security Availability Scalability	Naïve Bayes classifier	Collected SRs by interviews and other methods (255 FRs, NFRFR range from 10 to 67 per category)	NFR precision: 75%	Reduced manual labeling efforts.	Limited to NFR multi-class classification
[17] 2019	SR	FRs Availability Legal Look and feel Maintainability Operational Performance Scalability Security Usability Fault tolerance Portability	Multinomial naïve Bayes (MNB) Gaussian naïve Bayes (GNB) Bernoulli naïve Bayes (BNB) K-nearest neighbor (KNN) Support vector machine (SVM) Stochastic gradient descent SVM (SGD SVM) Decision tree (Dtree)	PROMISE dataset (625 requirement sentences: 255 FRs and 370 NFRs)	Precision: 0.66 Recall: 0.61 F1 score: 0.61 Accuracy: 0.76 Using SGD SVM classifier and TF-IDF for feature extraction	Seven algorithms used.	Need to apply other classification techniques such as boosting and bagging.
4. Complete System (Classifying NFRs and FRs into Multi-Classes)							
[20] 2020	1. SRs 2. NFRs 3. FRs	1. FRs NFRs 2. Usability Security Operational Performance 3. Functions Data Behavior	Fine-tuned BERT (NoRBERT)	PROMISE	1. F1 score: 90% FRs and 93% NFRs 2. F1 score: 76% 3. F1 score: 92%	Novelty in FR classification. Modern algorithm used with transfer learning.	Multi-class or multi-label classification for FRs not considered.

**Table 4:** PROMISE dataset details.

Classes	Number of Statements
Availability (A)	29
Fault tolerance (FT)	12
Legal (L)	14
Look and feel (LF)	42
Maintainability (MN)	17
Operational (O)	68
Performance (PE)	54
Portability (PO)	2
Scalability (SC)	21
Security (SE)	67
Usability (US)	67
FRs	266
Total	659

**Table 5:** Details of the PROMISE FR classes.

FR Classes	Number of Statements
Policy (P)	41
Solution (S)	116
Enablement (E)	71
Action constraint (AC)	25
Attribute constraint (AT)	7
Definition (D)	6
Total	266

**Table 6:** One-phase system: architecture of the BiLSTM DL model.

Layer	Output Shape	Number of Parameters
Embedding_64	(None, None, 46)	77,280
Spatial_dropout1d_39	(None, None, 46)	0
Bidirectional_39	(None, 92)	34,224
Dense_149	(None, 46)	4278
Dropout_95	(None, 46)	0
Dense_150	(None, 46)	2162
Dropout_96	(None, 46)	0
Dense_151	(None, 17)	799
Activation_39	(None, 17)	0

**Table 7:** One-phase system: Architecture of the CNN model.

Layer	Output Shape	Number of Parameters
Embedding_65	(None, None, 46)	77,280
Dropout_97	(None, None, 46)	0
Conv1d_10	(None, None, 46)	6394
Global_max_pooling1d_10	(None, 46)	0
Dense_152	(None, 128)	6016
Dropout_98	(None, 128)	0
Dense_153	(None, 17)	2193

**Table 8:** One-phase system: architecture of the GRU model.

Layer	Output Shape	Number of Parameters
Embedding_66	(None, None, 46)	77,280
Gru_17	(None, None, 46)	12,834
Gru_18	(None, 32)	7584
Dense_154	(None, 17)	561

**Table 9:** One-phase system: Architecture of the LSTM model.

Layer	Output Shape	Number of Parameters
Embedding_67	(None, None, 46)	77,280
Lstm_56	(None, None, 46)	17,112
Lstm_57	(None, 32)	10,112
Dense_155	(None, 17)	561

**Table 10:** One-phase system: evaluation parameters for the DL models.

DL Models	Accuracy (%)	Precision	Recall	F1 Score
BiLSTM	84.022	0.84	0.85	0.84
LSTM	74.656	0.75	0.74	0.72
CNN	91	0.92	0.92	0.92
GRU	78.78	0.79	0.79	0.78
Mean ensemble	88.15	0.88	0.89	0.88
Accuracy as a weight ensemble	90.63	0.91	0.91	0.91
Accuracy per class as a weight	92.56	0.93	0.93	0.93

**Table 11:** Two-phase system: architecture of the BiLSTM model in the binary classification phase.

Layer	Output Shape	Number of Parameters
Embedding_68	(None, None, 46)	60,858
Spatial_dropout1d_40	(None, None, 46)	0
Bidirectional_40	(None, 92)	34,224
Dense_156	(None, 46)	4278
Dropout_99	(None, 46)	0
Dense_157	(None, 46)	2162
Dropout_100	(None, 46)	0
Dense_158	(None, 3)	141
Activation_40	(None, 3)	0

**Table 12:** Two-phase system: architecture of the CNN model in the binary classification phase.

Layer	Output Shape	Number of Parameters
Embedding_69	(None, None, 46)	60,858
Dropout_101	(None, None, 46)	0
Conv1d_11	(None, None, 46)	6394
Global_max_pooling1d_11	(None, 46)	0
Dense_159	(None, 128)	6016
Dropout_102	(None, 128)	0
Dense_160	(None, 2)	258

**Table 13:** Two-phase system: architecture of the GRU model in the binary classification phase.

Layer	Output Shape	Number of Parameters
Embedding_70	(None, None, 46)	60,858
Gru_19	(None, None, 46)	12,834
Gru_20	(None, 32)	7584
Dense_161	(None, 2)	66

**Table 14:** Two-phase system: architecture of the LSTM model in the binary classification phase.

Layer	Output Shape	Number of Parameters
Embedding_71	(None, None, 46)	60,858
Lstm_59	(None, None, 46)	17,112
Lstm_60	(None, 32)	10,112
Dense_162	(None, 2)	66

**Table 15:** Two-phase system evaluation parameters for the DL models in the binary classification phase.

DL Models	Accuracy (%)	Precision	Recall	F1 Score
BiLSTM	95.00	0.94	0.95	0.94
LSTM	93.22	0.93	0.93	0.93
CNN	93.22	0.93	0.94	0.93
GRU	92.37	0.92	0.92	0.92
Mean ensemble	94.4	0.94	0.95	0.94
Accuracy as a weight ensemble	94.9	0.95	0.95	0.95
Accuracy per class as a weight ensemble	96	0.96	0.96	0.96

**Table 16:** Two-phase system: architecture of the BiLSTM model in the FR multi-class classification phase.

Layer	Output Shape	Number of Parameters
Embedding_8	(None, None, 46)	28,106
Spatial_dropout1d_3	(None, None, 46)	0
Bidirectional_3	(None, 92)	34,224
Dense_14	(None, 46)	4278
Dropout_9	(None, 46)	0
Dense_15	(None, 46)	2162
Dropout_10	(None, 46)	0
Dense_16	(None, 6)	282
Activation_3	(None, 6)	0

**Table 17:** Two-phase system: architecture of the CNN model in the FR multi-class classification phase.

Layer	Output Shape	Number of Parameters
Embedding_9	(None, None, 46)	28,106
Dropout1d_11	(None, None, 46)	0
Conv1d_3	(None, None, 46)	6394
Global_max_pooling1d_3	(None, 46)	0
Dense_17	(None, 128)	6016
Dropout_12	(None, 128)	0
Dense_18	(None, 6)	774

**Table 18:** Two-phase system: architecture of the GRU model in the FR multi-class classification phase.

Layer	Output Shape	Number of Parameters
Embedding_10	(None, None, 46)	28,106
GRU_5	(None, None, 46)	12,834
GRU_6	(None, 32)	7584
Dense_19	(None, 6)	198

**Table 19:** Two-phase system: architecture of the LSTM model in the FR multi-class classification phase.

Layer	Output Shape	Number of Parameters
Embedding_11	(None, None, 46)	28,106
Lstm_6	(None, None, 46)	17,112
Lstm_7	(None, 32)	10,112
Dense_20	(None, 6)	198

**Table 20:** Two-phase system: architecture of the BiLSTM model in the NFR multi-class classification phase.

Layer	Output Shape	Number of Parameters
Embedding_4	(None, None, 46)	53,958
Spatial_dropout1d_4	(None, None, 46)	0
Bidirectional_4	(None, 92)	34,224
Dense_21	(None, 46)	4278
Dropout_13	(None, 46)	0
Dense_22	(None, 46)	2162
Dropout_14	(None, 46)	0
Dense_23	(None, 11)	517
Activation_4	(None, 11)	0

**Table 21:** Two-phase system: architecture of the CNN model in the NFR multi-class classification phase.

Layer	Output Shape	Number of Parameters
Embedding_13	(None, None, 46)	53,958
Dropout_15	(None, None, 46)	0
Conv1d_4	(None, None, 46)	6394
Global_max_pooling1d_4	(None, 46)	0
Dense_24	(None, 128)	6016
Dropout_16	(None, 128)	0
Dense_25	(None, 11)	1419

**Table 22:** Two-phase system: architecture of the GRU model in the NFR multi-class classification phase.

Layer	Output Shape	Number of Parameters
Embedding_14	(None, None, 46)	53,958
GRU_7	(None, None, 46)	12,834
GRU_8	(None, 32)	7584
Dense_26	(None, 11)	363

**Table 23:** Two-phase system: architecture of the LSTM model in the NFR multi-class classification phase.

Layer	Output Shape	Number of Parameters
Embedding_15	(None, None, 46)	53,958
Lstm_9	(None, None, 46)	17,112
Lstm_10	(None, 32)	10,112
Dense_27	(None, 11)	363

**Table 24:** Two-phase system evaluation parameters of the DL classifiers for FRs in the multi-class classification phase.

DL Models	Accuracy (%)	Precision	Recall	F1 Score
BiLSTM	96.8	0.97	0.97	0.97
LSTM	78.91	0.79	0.79	0.79
CNN	96.0	0.96	0.96	0.96
GRU	58.59	0.59	0.59	0.59
Mean ensemble	96.8	0.97	0.97	0.97
Accuracy as a weight ensemble	96.8	0.97	0.97	0.97
Accuracy per class as a weight ensemble	98	0.98	0.98	0.98

**Table 25:** Two-phase system: evaluation parameters for the NFR DL classifiers in the multi-class classification phase.

DL Models	Accuracy (%)	Precision	Recall	F1 Score
BiLSTM	83.7	0.84	0.85	0.84
LSTM	63.0	0.64	0.63	0.64
CNN	83.3	0.83	0.83	0.83
GRU	56.0	0.57	0.56	0.55
Mean ensemble	85.0	0.85	0.84	0.85
Accuracy as a weight ensemble	85.1	0.85	0.85	0.85
Accuracy per class as a weight	86.5	0.89	0.86	0.87

**Table 26:** Two-phase classification system: Evaluation parameters of all phases together.

DL Models	Average Accuracy (%)	Average Precision	Average Recall	Average F1 Score
BiLSTM	91.66	0.91	0.92	0.91
LSTM	78.37	0.78	0.78	0.78
CNN	90.84	0.90	0.91	0.90
GRU	68.98	0.69	0.69	0.68
Mean ensemble	92.06	0.92	0.92	0.92
Accuracy as a weight ensemble	92.26	0.92	0.92	0.92
Accuracy per class as a weight	93.4	0.94	0.93	0.93

**Table 27:** Comparison of the proposed system with other state-of-the-art systems.

Reference #	Classification		Methodology	Dataset	Results
Year	Input	Classes			
1. Classifying NFRs into Multi-Classes					
[7] 2017	SRs	Functional Availability	CNN	PROMISE corpus	Precession: 0.80
		Legal			Recall: 0.785
		Look and feel Maintainability Operational			F measure: 0.77
		Performance Scalability			
		Security			
		Usability			
		Fault tolerance Portability			
[18] 2018 1. SR 2. NFRs 3. NFRs	1. FRs NFRs 2. Availability Legal Maintainability Operational Performance Scalability Look and feel Security Usability 3. Security Non-security	CNN	PROM corpus	1. F1 score: 0.945 2. F1 score: 0.911 3. F1 score: 0.772	
[17] 2019	SRs	FR Availability Legal Look and feel Maintainability Operational Performance Scalability Security Usability Fault tolerance Portability	Multinomial naïve Bayes (MNB) Gaussian naïve Bayes (GNB) Bernoulli naïve Bayes (BNB) K-nearest neighbor (KNN) Support vector machine (SVM) Stochastic gradient descent SVM (SGD SVM) Decision tree (Dtree)	PROMISE dataset	Precision: 0.66 Recall: 0.61 F1 score: 0.61 Accuracy: 0.76 Using the SGD SVM classifier and TF-IDF for feature extraction
2. Complete System (classifying NFRs and FRs into Multi-Classes)					
[20] 2020	1. SRs 2. NFRs 3. FRs	1. FRs–NFRs 2. Usability Security Operational Performance 3. ions Data behavior	Fine-tuned BERT (NoRBERT)	PROMISE	1. F1 Score: 90% FRs and 93% NFRs 2. F1 Score: 76% 3. F1 Score: 92%
2021 Proposed one-phase classification system	SRs	17 classes of FRs and NFRs (6 classes of FRs and 11 classes of NFRs)	Ensemble DL-based model (BiLSTM-LSTM-CNN-GRU)	PROMISE	Accuracy: 92.56% Precision: 0.93 Recall: 0.93 F1 Score: 0.93
2021 Proposed two-phase classification system	SRs	1. Phase one: Binary classification into FRs or NFRs 2. Phase two: Multi-class classification of FRs (6 classes) and NFRs (11 classes)	Ensemble DL-based model (BiLSTM–LSTM–CNN–GRU)	PROMISE	1.Accuracy: 95.7% Precision: 0.96 Recall: 0.96 F1 Score: 0.96 2. Accuracy: 93.4% Precision: 0.94 Recall: 0.93 F1 Score: 0.93

## Data Availability

Not applicable.
